# Bacterial Re-Colonization Occurs Early after Lung Transplantation in Cystic Fibrosis Patients

**DOI:** 10.3390/jcm10061275

**Published:** 2021-03-19

**Authors:** Anna Engell Holm, Hans Henrik Lawaetz Schultz, Helle Krogh Johansen, Tania Pressler, Thomas Kromann Lund, Martin Iversen, Michael Perch

**Affiliations:** 1 Department of Cardiology, Section for Lung Transplantation, Copenhagen University Hospital, Rigshospitalet, 2100 Copenhagen, Denmark; Annaengellholm@gmail.com (A.E.H.); Thomas.kromann.lund@regionh.dk (T.K.L.); martin@iversen-net.dk (M.I.); michael.perch@regionh.dk (M.P.); 2 Department of Clinical Microbiology, Copenhagen University Hospital, Rigshospitalet, 2100 Copenhagen, Denmark; hkj@biosustain.dtu.dk; 3 Department of Clinical Medicine, Faculty of Health and Medical Sciences, University of Copenhagen, 2100 Copenhagen, Denmark; 4 Department of Infectious Disease, Cystic Fibrosis Centre, Copenhagen University Hospital, Rigshospitalet, 2100 Copenhagen, Denmark; tacjana.pressler@regionh.dk

**Keywords:** cystic fibrosis, lung transplantation, colonization

## Abstract

Most cystic fibrosis (CF) patients referred for lung transplantation are chronically infected with Gram-negative opportunistic pathogens. It is well known that chronic infections in CF patients have a significant impact on lung-function decline and survival before transplantation. The rate and timing of re-colonization after transplantation have been described, but the impact on survival after stratification of bacteria is not well elucidated. We did a single-center retrospective analysis of 99 consecutive CF patients who underwent lung transplantation since the beginning of the Copenhagen Lung Transplant program in 1992 until October 2014. Two patients were excluded due to re-transplantation. From the time of CF diagnosis, patients had monthly sputum cultures. After transplantation, CF-patients had bronchoscopy with bronchoalveolar lavage at 2, 4, 6 and 12 weeks and 6, 12, 18 and 24 months after transplantation, as well as sputum samples if relevant. Selected culture results prior to and after transplantation were stored. We focused on colonization with the most frequent bacteria: *Pseudomonas aeruginosa* (PA), *Stenotrophomonas maltophilia* (SM), *Achromobacter xylosoxidans* (AX) and *Burkholderia cepacia complex* (BCC). Pulsed-field gel electrophoresis (PFGE) was used to identify clonality of bacterial isolates obtained before and after lung transplantation. Time to re-colonization was defined as the time from transplantation to the first positive culture with the same species. Seventy-three out of 97 (75%) had sufficient culture data for analyses with a median of 7 (1–91) cultures available before and after transplantation. Median colonization-free survival time was 23 days until the first positive culture after transplantation. After 2 years, 59 patients (81%) were re-colonized, 33 (48.5%) with PA, 7 (10.3%) with SM, 12 (17.6%) with AX, and 7 (10.3%) with BCC. No difference in survival was observed between the patients colonized within the first 2 years and those not colonized. Re-colonization of bacteria in the lower airways occurred at a median of 23 days after transplantation in our cohort. In our patient cohort, survival was not influenced by re-colonization or bacterial species.

## 1. Introduction

Lung transplantation is a well-established treatment for selected patients with end-stage cystic fibrosis (CF). CF is the most common indication for transplantation among younger recipients [1]. A major challenge when transplanting CF patients is the presence of microbial pathogens at the time of transplant, increasing the risk for infectious complications in the early post-operative period as well as later recurrence in the transplanted lung in part due to immunosuppressants [2,3,4,5]. This has been shown for specific pathogens like *Burkholderia cepacia complex* (BCC) [6], which is a risk factor for developing pneumonia [7] and death in the early post-transplant period where immunosuppression is at maximum [8,9,10].

Chronic airway infection with pan-resistant *Pseudomonas aeruginosa* [10], non-epidemic *Burkholderia cenocepacia*, *Burkholderia gladioli* [11] and *Mycobacterium abscessus* prior to transplantation leads to a higher risk of re-colonization and a suggested increased risk of morbidity and mortality [8,12].

The primary cause of morbidity and mortality after lung transplantation is chronic lung allograft dysfunction (CLAD), affecting up to 50% of all patients after 5 years [13,14]. The rate of colonization and its association with CLAD and best-achieved pulmonary functions (FEV_1_, FVC and diffusion capacity) after transplantation is not well elucidated [15]. However, a link has been shown in a previous single-center study, which found that colonization after transplantation was independently associated with increased risk of CLAD [16]. The use of pulsed-field gel electrophoresis (PFGE) to investigate the relatedness of the bacteria found before and after lung transplantation has not previously been done.

The aim of this study was

(1) to investigate the rate of re-colonization stratified by type of bacteria;
(2) to demonstrate whether bacteria were genetically identical before and after lung transplantation by the use of PFGE;
(3) to investigate the impact of infection on CLAD, pulmonary function and survival.

## 2. Materials and Methods

We did a single-center retrospective study of all double lung transplanted CF-patients in Denmark since the beginning of the lung transplant program in 1992 until October 2014. All Danish lung transplantations are centralized at Rigshospitalet, Copenhagen, Denmark.

The analysis focused on the most frequently cultured bacteria *Pseudomonas aeruginosa* [17,18] (PA), *Stenotrophomonas maltophilia* (SM), *Achromobacter xylosoxidans* (AX) and *Burkholderia cepacia complex* (BCC). All CF patients were followed before and after lung transplantation, at one of the two Danish Cystic Fibrosis Centers and at the national lung transplant center, and all clinical and microbiological cultures were collected from the CF-database and from the microbiology data system (MADS) at Department of Clinical Microbiology at Rigshospitalet.

### 2.1. Pharmacological Treatment after Lung Transplantation

All recipients received induction therapy with either ATG or Daclizumab at transplantation. Lifelong maintenance immunosuppression therapy was a combination of Cyclosporine, Azathioprine and steroid.

All patients received some combination of systemic antimicrobial treatment with at least two drugs for the first couple of weeks after the transplant procedure. This was combined with antifungal and antiviral prophylaxis, which was administered during the first 12 weeks.

### 2.2. Bronchoscopy

The lung transplant surveillance program includes bronchoscopy with bronchoalveolar lavage (BAL) sampling at 2, 4, 6 and 12 weeks and at 6, 12, 18 and 24 months after transplantation as well as additional bronchoscopy if clinically relevant, e.g., in the case of acute unexplained drop in lung function.

BAL was performed with three aliquots of 50 mL with the bronchoscope wedged into the middle lobe or lingula. Samples were stored at room temperature before shipment to the department of clinical microbiology within six hours.

### 2.3. Microbiology

In the transplanted CF population, BAL fluid is routinely cultured for CF-bacteria and fungi at all time points. All BAL fluids and airway secretions were Gram-stained to ensure the origin from the lower airways and cultured on selective media in the clinical microbiological laboratory. These media included a Sabouraud plate, a 7% NaCl plate, a *B. cepacia* plate containing colistin and gentamicin and a “blueplate” (modified Conradi Drigalski’s medium) selective for Gram-negative rods and non-selective media including 5% Danish blood agar and chocolate agar (Statens Serum Institute, Copenhagen, Denmark). Before 2011, isolated bacteria were identified using biochemical profiling based on API 20NE (bioMérieux), and from 2011, MALDI-TOF MS (Bruker Daltronics, Bremen Germany) was used [19].

Date of colonization after transplantation was defined as the first positive BAL culture, out of at least two positive BAL cultures including the same micro-organism, respectively. Re-colonization was defined as colonization after lung transplantation with the same type of bacteria as before transplantation.

### 2.4. Bacteria Isolates

Since the beginning of the nineties, *P. aeruginosa*, and later on *S. maltophilia*, *A. xylosoxidans, B. cepacia complex* and *P. apista* isolates from sputum were collected and stored (−80 °C) 20 annually.

### 2.5. Pulsed-Field-Gel-Electrophoresis (PFGE)

PFGE was used for the identification of clonality of bacterial isolates obtained before and after lung transplantation. We used Spe I as a restriction enzyme for all typed isolates (*P. aeruginosa, S. maltophilia, A. xylosoxidans, B. cepacia complex* and *P. apista*) according to methods described previously [19,20,21]. PFGE patterns were compared visually and evaluated using the criteria previously described [21,22]. Isolates with differences in zero, one or two bands were considered identical; other isolates were considered unrelated.

### 2.6. Lung Function Tests

The patient’s lung function was closely monitored with per-protocol spirometry at least every third month at the lung transplantation clinic as well as body box plethysmography at 3, 6, and 12 months and yearly after the transplantation. If the patient experienced a decline in absolute FEV_1_, new pulmonary tests were made. The static lung volumes were measured using spirometry lung-function tests calculating the expiratory flow rates, and using body box plethysmography, dynamic and static lung volumes were measured.

### 2.7. Chronic Lung Allograft Dysfunction

Baseline FEV_1_ and forced vital capacity (FVC) post-transplant were defined as the average of the 2 best lung-function tests at least three weeks apart according to the standard criteria for International Society for Heart and Lung Transplantation (ISHLT) [23]. Baseline diffusion capacity for carbon monoxide (DL_CO_), residual volume (RV) and total lung capacity (TLC) was defined as the measured value at the same time or first after the baseline values for FEV_1_ and FVC, if patients did not reach the threshold for CLAD. CLAD was defined as a persistent decrease in FEV_1_ < 80% of baseline value with or without an associated decrease in TLC < 90% of baseline value, also conditional on 3 months survival according to standard ISHLT criteria.

Survival was calculated as the time of transplantation to the time of death or by censoring the patients when data were updated in October 2014.

### 2.8. Statistics

Statistical analysis was performed using IBM SPSS Statistics (version 22.0) software. Kaplan–Meier plots with Log-Rank tests were employed to demonstrate survival and CLAD-free survival, while stratifying for types of microbes. Continuous variables were described as mean ± standard deviation (±SD) and compared using ANOVA. A *p*-value ≤ 0.05 was considered statistically significant.

## 3. Results

**Figure 1:**
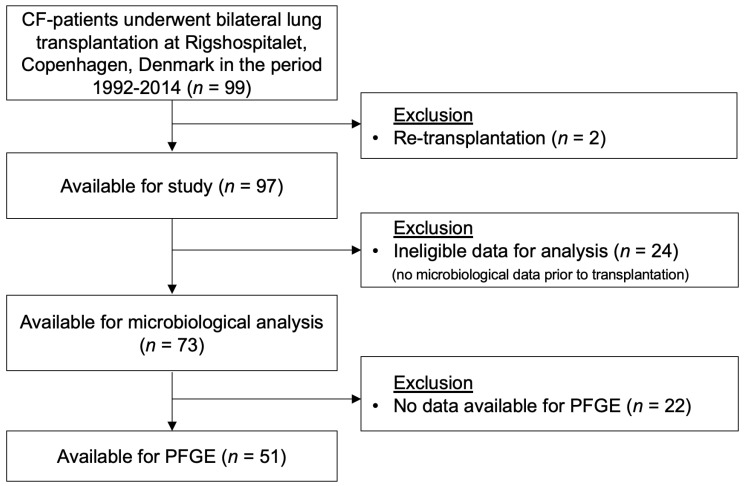
This diagram shows how patients were included and excluded in this study.

During the period from 1992 to 2014, 99 CF patients underwent bilateral lung transplantation at Rigshospitalet, Copenhagen, Denmark. As described in flowchart 1, two of the patients were excluded from the follow-up study due to re-transplantation. In total, 73 out of 97 patients (75%) had sufficient data for inclusion in the study. See Figure 1 for patient inclusion and exclusion. If there were no bacterial isolates prior to transplantation or frozen cultures available for PFGE, patients were excluded for these analyses; see Table 1 for demography.

### 3.1. Re-Colonization

The re-colonization after transplantation stratified by type of bacteria present in BAL fluid was investigated. We found that *P. aeruginosa* (*n* = 39), *S. malthophilia* (*n* = 7), *A. xylosoxidans* (*n* = 12) and *B. cepacia complex* (*n* = 7) were the most frequently cultured bacteria. Eight patients did not experience re-colonization during the study period. There was no significant difference in outcome between the patients stratified by re-colonization when comparing age at transplantation.

**Figure 2:**
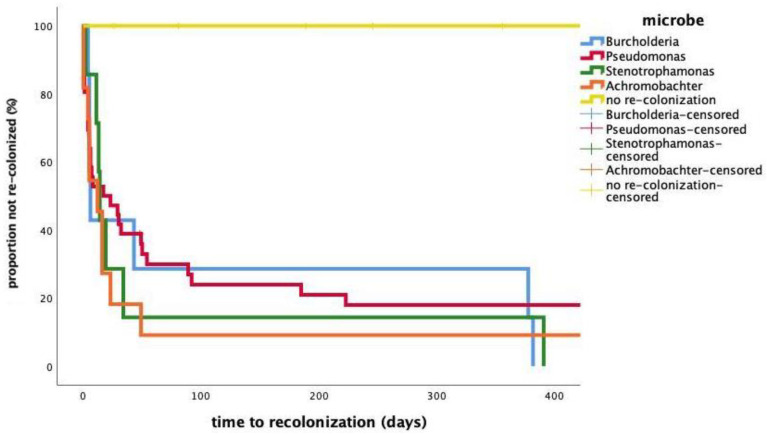
Kaplan–Meyer plot showing time from transplantation to re-colonization.

The median time passed to re-colonization after transplantation in all patients was 23 days. The colonization-free survival after transplantation stratified by bacteria is depicted in Figure 2. No difference in the rate of re-colonization was observed between all types of microbes using Log-Rank test (Mantel-Cox) (*p* = 0.46).

### 3.2. Pulsed-Field Gel Electrophoresis

**Figure 3:**
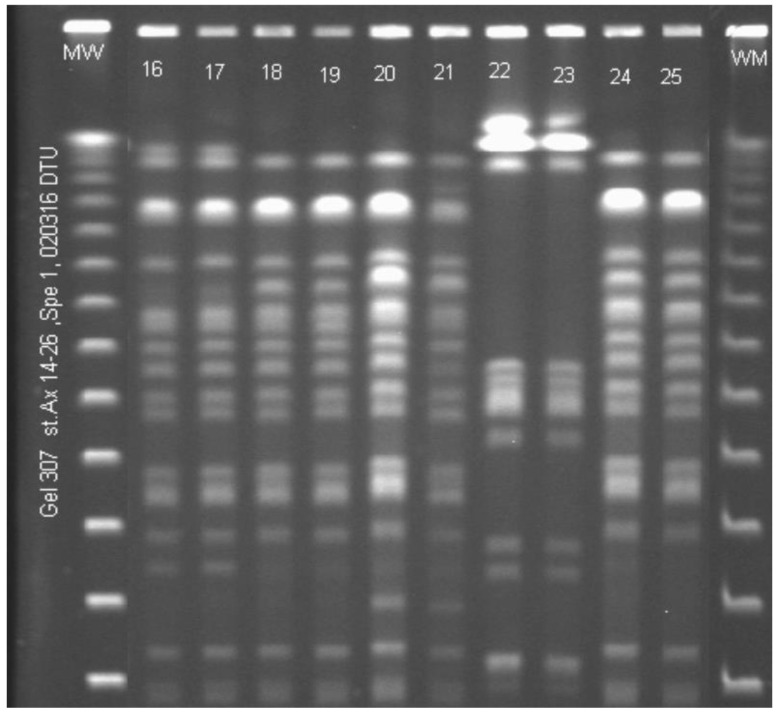
Pulsed-field-gel-electrophoresis pattern of 10 paired Achromobacter xylosoxidans isolates from bronchoalveolar lavage (BAL) fluid from five cystic fibrosis (CF) patients before and after lung transplantation (ltx). The Spe restriction enzyme was used. MW: molecular-weight standard. Lane 16 and 17: CF334 ML, before (lane16) and after ltx (lane 17). Lane 18 and 19: CF340 JP, before (lane 18) and after ltx (lane 19). Lane 20 and 21: CF353 ST, before (lane 20) and after ltx (lane 21). Lane 22 and 23: CF172 TN, before (lane 22) and after ltx (lane 23). Lane 24 and 25: CF347 HM, before (lane 24) and after ltx (lane 25).

Out of 73 lung transplantation recipients, 51 patients had pre-transplant isolates available for PFGE and were colonized with the same bacteria after transplantation. These bacteria were distributed on the following pathogens: 38 patients colonized with *P. aeruginosa*, two with *S. maltophilia*, five with *A. xylosoxidans* (Figure 3), five with *B. cepacia complex* and one with *P. apista*.

We examined the PFGE pattern among these pre- and post-transplant isolates and found that twenty-four out of the 38 patients (63%) who had chronic *P. aeruginosa* infection had an identical PFGE pattern pre- and post-lung-transplantation. All bacterial isolates from patients with chronic *S. maltophilia, A. xylosoxidans, B. cepacia complex* and *P. apista* had the same PFGE pattern before and after lung transplantation.

The median time between isolates collected pre- and post-lung-transplantation was 2 years (range 1–16 years) in *P. aeruginosa* infected patients with bacteria having the same PFGE pattern compared to 3 years (range 1–12 years) in patients with different PFGE pattern.

### 3.3. Impact of Colonization on Mortality

**Figure 4:**
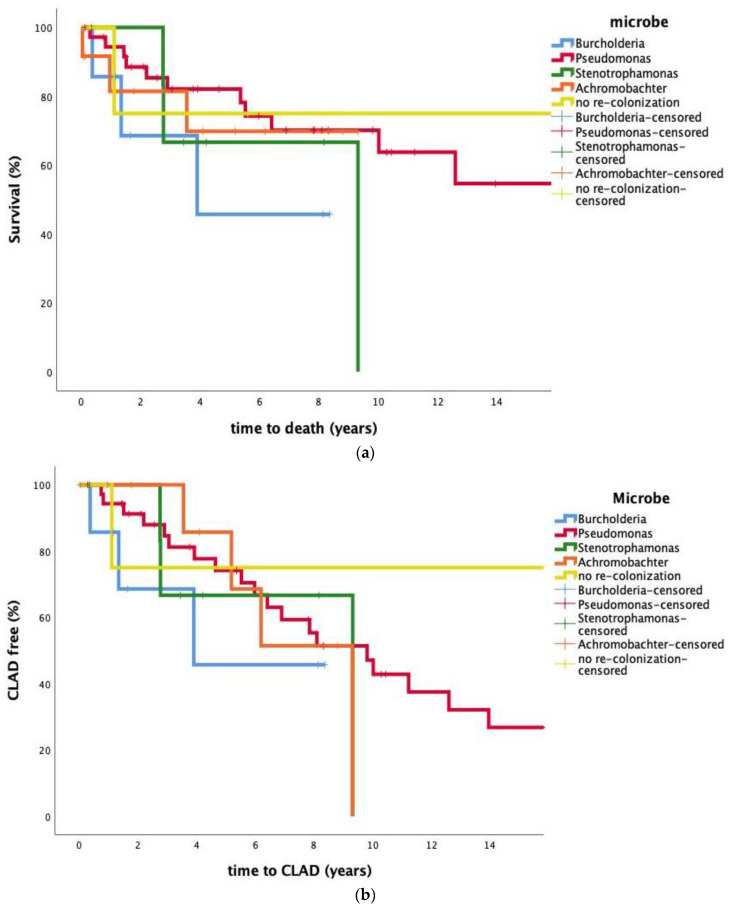
(**a**,**b**) demonstrate the time CLAD and death after transplantation stratified for different bacteria.

The impact of re-colonization on survival was analyzed, stratifying for bacterial species colonization of the patients. Median survival times were 13.7 years for *P. aeruginosa*, 7.1 years for *S. maltophilia*, 7.0 years for *A. xylosoxidans* and 5.0 years for *B. cepacia complex*. Kaplan–Meier plots are shown in Figure 4a. No statistically significant difference in survival was observed within the four bacterial subgroups of colonized patients; however, a statistically significant difference was present when comparing non-colonized patients to *B. cepacia* complex Log-Rank (*p* = 0.029), *P. aeruginosa* Log-Rank (*p* < 0.001) and *A. xylosoxidans* Log-Rank (*p* = 0.007) but not when comparing the non-colonized to *S. maltophilia* Log-Rank (*p* = 0.106).

### 3.4. Impact of Colonization on CLAD

The impact of re-colonization on CLAD-free survival was investigated and stratified by bacteria. We found an estimated median CLAD free survival time of 10.0 years for *P. aeruginosa*, 7.1 years for *S. maltophilia* and 7.2 years for *A. xylosoxidans*. Two out of six patients who were re-colonized with *B. cepacia complex* encountered CLAD. A Kaplan–Meier plot showing CLAD-free survival is depicted in Figure 4b. No statistically significant difference in CLAD free survival was observed when comparing the four colonized groups’ Log-Rank (Mantel-Cox) (*p* = 0.752). Estimation was limited to the largest follow-up time if it was censored.

## 4. Discussion

Re-colonization of the lower airways after lung transplantation in CF patients is a well-known problem, and it is a major contributor to increased hospitalizations and the use of antibiotics [7]. The finding that the time to re-colonization was only 23 days post-transplantation corroborates findings from the early days of lung transplantation, where *P. aeruginosa* was found merely 15 days post-transplantation in a CF population 18.

The rate of colonization and its impact on survival in patients infected with, *P. aeruginosa*, *S. maltophilia* [24] *A. xylosoxidans* [25] and *B. cepacia complex* [25,26] has been described earlier, but its influence on post-transplant outcomes such as survival free of CLAD and impact on absolute values of baseline pulmonary function is not well described. One study found de novo and persistent colonization with *P. aeruginosa* to correlate with development of CLAD [27] compared to non-colonization.

Using Log Rank-test (Mantel-Cox), we found no difference in the rate of re-colonization between the different bacteria. The early re-colonization is likely due to aspiration from the upper airways, and it has previously been shown that most CF patients chronically infected with *P. aeruginosa* have the clone type in the upper and lower airways because of bi-directional communication [28,29].

The use of PFGE typing to investigate the relatedness of the bacteria found before and after lung transplantation has not previously been done. With our analysis, we found that all patients infected with *S. maltophilia*, *A. xylosoxidans*, *B. cepacia complex* and *P. apista* had the same PFGE pattern before and after lung transplantation. However, only 63% of the patients chronically infected with *P. aeruginosa* had the same PFGE pattern as before transplantation.

When looking at the impact of re-colonization on CLAD and mortality, we found no significant difference in CLAD-free survival when stratified for bacteria, and indeed some patients with early-onset CLAD were not re-colonized.

When we looked at re-colonization and its impact on survival stratifying for colonization status of the patients, we found no significant difference in survival within the four subgroups of colonized patients; however, a statistically significant difference was present when comparing non-colonized patients to *B. cepacia complex*, *P. aeruginosa* and *A. xylosoxidans*. This result could be caused by non-colonized patients that die before a potential re-colonization is detected. Our results are consistent with the results by Srour N et al. [30], who investigated pre-transplantation colonization of the lower airways with transmissible strains of *P. aeruginosa* in CF patients and its impact on morbidity and mortality post-transplantation. The study found that pre-transplant colonization was not associated with CLAD-free survival, frequency of acute rejection, frequency of post-transplant respiratory infection or post-transplant decline in FEV_1_.

The results of our study suggest that re-colonization after lung-transplantation in CF patients has no impact on the patient’s post-transplant morbidity and mortality. As nearly all patients were re-colonized early, it was not possible to determine the effect of recolonization per se.

### Limitations

A major limitation is the retrospective study design and small number of patients and available data, thus yielding low statistical power. This is because of a limited number of CF patients in Denmark and a similarly limited need for transplantation. Because of the long inclusion period of 22 years, changes in the choice of therapy and patient care could have biased our findings, specifically when comparing newer patients with historical groups. Donor organ quality in the entire era has been investigated and published previously [31,32].

We used PFGE as a way of assessing the epidemiological relationship between most common bacterial infections. Even though PFGE is a gold standard method, it has its limitations. The method cannot detect every genetic change; however, it is estimated that the average bacterial PGFE pattern represents more than 90% of the total genome [33].

## 5. Conclusions

In conclusion, we demonstrated that re-colonization of CF patients with the same bacterial strain is very common and happens early after transplantation, with a median time of 23 days. The rate of re-colonization with *B. cepacia complex*, *P. aeruginosa*, *S. maltophilia* and *A. xylosoxidans* was similar, and it did not seem to impact survival or CLAD-free survival, although numbers were small. The absolute values of pulmonary function both at baseline and at CLAD were not influenced by colonization. As this was a single-center study, with only a few patients included, our results should be investigated in future and larger clinical prospective studies. However, the results from this single-center study has resulted in a change in the post-transplant procedure for CF patients at our institution. The rate of re-colonization was earlier than expected, and even though the study found no impact on survival, other studies have found a higher risk for infectious complications in the early post-operative period as well as later recurrence in the transplanted lung, in part due to immunosuppressants [2,3,4,5]. The new standard practice includes a functional endoscopic sinus surgery 2–3 weeks after transplantation under wide antibiotic cover.

## Figures and Tables

**Table 1:** Demography of the patients including lung function at baseline and at chronic lung allograft dysfunction (CLAD). Variables are presented as median and standard deviation.

	Burkholderia (*n* = 7)	Pseudomonas (*n* = 39)	Stenotrophamonas (*n* = 7)	Achromobachter (*n* = 12)	Non-Recolonization (*n* = 8)	*p*-Value
Gender female (%)	6/7	21/39	2/7	4/12	3/8	0.702
Age at transplantation (years)	28.7 (±7.8)	31.2 (±7.4)	39.1 (±10.7)	27.3 (±9.9)	22.3 (±9.2)	0.162
Donor Pulse (BPM)	112 (±30.1)	99.5 (±28.0)	100 (±17.2)	90 (±49.7)	99.5 (±12.7)	0.997
Donor Systolic BP (mmHg)	122 (±15.9)	114 (±25.5)	109 (±17.7)	111 (±26.7)	121 (±12.9)	0.941
Donor Diastolic BP (mmHg)	71.5 (±18.8)	65 (±16.3)	71 (±15.8)	62.5 (±14.0)	81.5 (±13.9)	0.575
Donor Weight (Kg)	65.0 (±7.4)	65 (±12.5)	67.5 (±18.9)	76.5 (±12.5)	69.0 (±8.12)	0.542
Donor Height (m)	1.67 (±0.11)	1.71 (±0.07)	1.78 (±0.11)	1.70 (±0.10)	1.70 (±0.11)	0.228
Donor age (years)	43.0 (±9.5)	43.0 (±12.5)	37.0 (±16.8)	42.0 (±15.7)	41.0 (±18.2)	0.832
Baseline FVC (L)	3.23 (±0.81)	3.40 (±0.67)	4.08 (±1.34)	3.74 (±1.38)	3.9 (±0.95)	0.215
Baseline FEV_1_ (L)	2.56 (±0.56)	2.62 (±0.66)	3.70 (±1.11)	3.05 (±1.0)	2.98 (±0.9)	0.179
Baseline RV (L)	1.7 (±0.20)	1.95 (±0.74)	2.28 (±0.50)	1.82 (±0.55)	1.56 (±0.85)	0.441
Baseline TLC (L)	5.1 (±0.84)	5.42 (±1.43)	6.35 (±1.72)	4.86 (±1.97)	5.49 1.80	0.364
Baseline DL_CO_	6.1 (±0.78)	5.95 (±1.58)	6.6 (±2.3)	5.38 (±1.54)	5.52 (±2.1)	0.701
Baseline DL_CO_ alveolar	1.1 (±0.23)	1.26 (±0.20)	1.16 (±0.16)	1.12 (±0.21)	1.11 (±0.29)	0.640
